# Scientific Items

**Published:** 1884-06-20

**Authors:** 


### SCIENTIFIC ITEMS.

**The "Dugong," or Vegetarian Whale.**—A writer in the Gentleman's Magazine gives some interesting particulars relative to this species of whale, now taken, to a considerable extent, in Queensland, and valuable alike for its oil and its food. Its size varies from eight to twenty feet in length; it lives upon submarine meadows of seaweed; It has no gills, but breathes air by means of lungs; its head is round and somewhat humanlike, and has hair something like that of a man's beard. It is said that many stories of merman and mermaid may be traced to these creatures. Their oil is said to have all the medicinal merits of cod-liver oil, without its unpleasant flavor; at ordinary temperature it deposits crystals, as olive-oil does in frosty weather, but on warming slightly becomes liquid and clear. The flesh is much prized in Australia, being cut off in flitches and slabs, and it is stated that "from the same animal is taken meat resembling beef, veal, and bacon."

**The Balloon as a Telegraph Medium.**—A method of signalling by means of electric balloons has been successfully tried in Paris by MM. Mangin and Baudet. The balloon, made of paper rendered translucent, was about eight feet in diameter, and was filled with pure hydrogen. A Swan lamp was fitted inside; and a light rope carrying two copper wires was attached. When the circuit was completed, the whole balloon appeared to be a globe of fire. By switching the current off and on, the Morse code can be spelled out by which communication can be carried on between distant points.—*Pop. Science News.*

**Pasteur's Laboratory.**—The following cheerful description of M. Pasteur's laboratory is taken from a recently published volume entitled L'Histoire a'um Savant par un Ignorant : All the animals in the laboratory, from the little white mice hiding under a bundle of cotton-wool, to the dogs barking furiously from behind their iron-railed kennels, are doomed to death. These inhabitants of the laboratory, which are marched out day after day in order to be subjected to operations or other experiments, share the space with still more ghastly objects. From all parts of France, hampers arrive containing fowls which have died of cholera or some other disease. Here is an enormous basket bound with straw; it contains the body of a pig which has died of fever. A fragment of lung, forwarded in a tin box, is from a cow dying of pneumonia. Other goods are still more precious. Since M. Pasteur, two years ago, went to Pauillac to await the arrival of a boat which brought yellow fever patients, he recieves now and then from far off countries a bottle of vomito negro. Tubes filled with blood are lying about; and small plates containing drops of blood may be seen everywhere on the work-tables. In special stores, bottle-like bladders are ranged resembling small liquor-bottles. The prick of a pin into one of these bladders would bring death to any man. Enclosed in glass prisons millions and millions of microbes live and multiply.—*Pop. Science News.*

**The Moon and the Weather.**—Prof. Young, (Pop. Science News) remarks:

Since the superstition as to the moon's influence upon the wind and weather, is so wide spread and deep seated, a word on that subject may be in order. In the first place, since the total heat received from the moon, even according to the highest determination (that of Smyth), is not so much as 1-100000 of that received from the sun, and since the only hold the moon has on the earth's weather is through the heat she sends us (I ignore here the utterly insignificant atmospheric tide), it follows necessarily that her influence must be very trifling. In the next place, all carefully collected observations show that it is so, and not only trifling, but generally absolutely insensible.

For example, different investigators have examined the question of nocturnal cloudiness at the time of full moon, there being a prevalent belief that the full moon "eats up" light clouds. On comparing thirty or forty years' observations at each of several stations (Greenwich, Paris, etc.), it is found that there is no ground for the belief. And so in almost every case of imagined lunar meteorological influence. As to the coincidence of weather-changes with changes of the moon, it is enough to say that the idea is absolutely inconsistent with that progressive movement of the "weather" across the country from west to east, with which the Signal Service has now made us all so familiar.

**Photographic Progress.**—Photography is one of the miracles of modern times. The art has taken strides with seven-league boots since the time of Daguerre, who made a picture on a metal plate, which had to be turned and twisted in every direction before you could find what you were looking after. Lately pictures have been taken with such rapidity, that lightning is compelled to strike a faster gait to keep up with the process. Dr. Koch tells us that he has photographed with a photomicrographic camera that most minute filament, the flagellum of the bacteria. One might be satisfied with photographing the bacteria as a whole, since it is beyond the reach of naked vision; but to take a picture of an insignificant portion of it is like getting among fairy-tales. Mr. Rockwood, also, has succeeded in getting satisfactory pictures of the vibrating-point in the diaphragm of a telephonic instrument while a person was speaking at it, and by means of a spark from Leyden-jars. The work must have been done in the one twenty-four thousandth part of a second.—*Pop. Science News.*

